# Automatic Control of Arterial Carbon Dioxide Tension in Mechanically Ventilated Patients

**DOI:** 10.1109/TITB.2002.806084

**Published:** 2002-12-10

**Authors:** Tyrone Fernando, John Cade, John Packer

**Affiliations:** 1 Department of Electrical and Electronic EngineeringUniversity of Western Australia Crawley WA 6009 Australia; 2 Intensive Care UnitRoyal Melbourne Hospital Parkville 3052 Australia; 3 Department of Electrical and Electronic EngineeringUniversity of Melbourne Parkville 3052 Australia

**Keywords:** Arterial carbon dioxide tension, automatic control, mechanical ventilation

## Abstract

This paper presents a method of controlling the arterial carbon dioxide tension of patients receiving mechanical ventilation. Controlling of the CO_2_ tension is achieved by regulating the ventilator initiated breath frequency and also volume per breath.

## I. Introduction

Modern lung ventilators are equipped with a variety of support techniques that provide the clinician various ways of artificially ventilating patients. Mandatory minute ventilation (MMV) is one such ventilation technique that interact with the patient in such a way that the delivery of a preset minimum minute volume (inhaled volume per minute) is guaranteed. The number of volume control breaths^1.^^1.^A volume control breath delivers a fixed volume specified by an operator. per minute automatically increases or decreases to ensure the delivery of the requested mandatory or minimum minute volume. The concept of MMV appears attractive in that it provides the ability to increase or decrease the number of volume control breaths in a given time span. Thus, if the patient is apneic, an appropriate number of volume control breaths are delivered, and as the patient resumes spontaneous ventilation, the number of volume control breaths decreases gradually. This support scheme provides flexibility for a patient to automatically take up the work load of breathing and for the machine to gradually wind down at the same time.

For a patient ventilated in MMV, the clinician selects the level of minimum minute ventilation and this setting remains unaltered until a change is requested by the clinician. In the event of an increase or decrease in arterial CO_2_ tension, the MMV setting may be decreased or increased respectively by the clinician to correct the disturbed arterial CO_2_ tension. Regulating the MMV setting to maintain an acceptable arterial CO_2_ tension could be performed automatically. This paper deals with such an automatic ventilation scheme that maintains the arterial CO_2_ tension less than a specified level determined by a clinician.

**Fig. 1.**
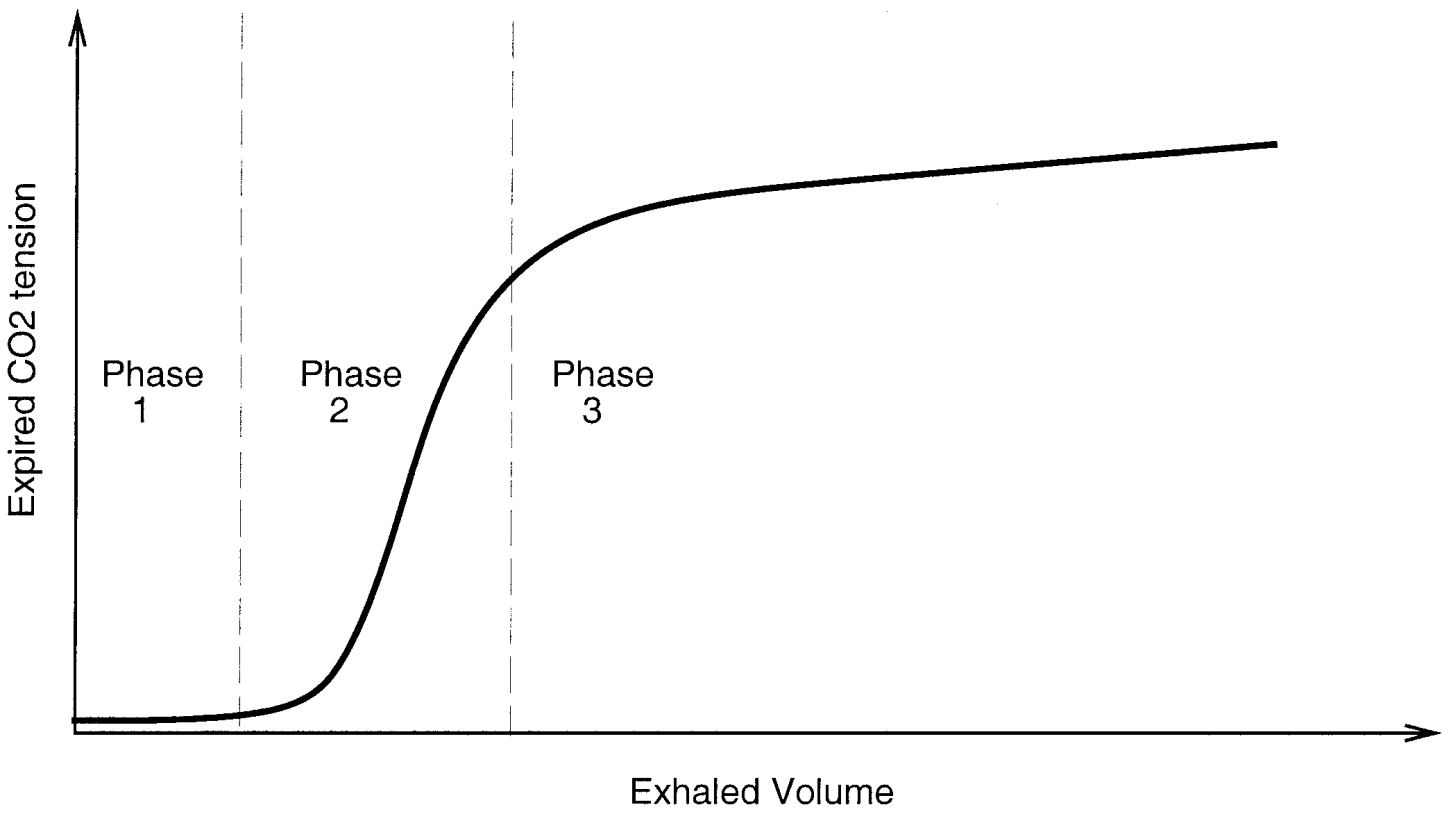
Three phases of SBT-CO_2_ plot.

## II. Respiratory Dead Space

During respiration not all inhaled air reaches the alveoli, as part of it remains in the conducting portion of the airway. The region of the respiratory system where gas exchange does not take place is termed the respiratory dead space. By examining the CO_2_ content of the exhaled air, Bohr formulated the following relationship [1]:

$${{V_{T}-V_{D}}\over {V_{T}}} = {{P_{\,\overline{E}_{{\rm CO}_2}}}\over {P_{a_{{\rm CO}_2}}}}\eqno{\hbox{(1)}}$$

where $V_{D}$ is the dead space, $V_{T}$ is the tidal volume (i.e., volume inhaled per breath), $P_{a_{{\rm CO}_2}}$ is the arterial CO_2_ tension, and $P_{\,\overline{E}_{{\rm CO}_2}}$ is the average CO_2_ tension in exhaled air. A typical trace of exhaled CO_2_ tension against exhaled volume (i.e., SBT-CO_2_ plot^2.^^2.^Single breath test of carbon dioxide.) consists of three phases [1]: phase 1 comprising the CO_2_-free gas from the airways, phase 2 a transition phase characterized by a S-shaped up-swing in the tracing representing the washout of airway with alveolar gas, and phase3 comprising CO_2_-bearing gas from the alveoli (see Fig. 1). The initial volume of expirate which includes the volume of fresh air down to the alveolar/fresh air interface represents the airway dead space. Airway dead space is, therefore, defined as the volume of air that does not reach the sites of gas exchange (i.e., alveolus). On the SBT-CO_2_ plot this corresponds to an exhaled volume in phase 2. The exact location of this position on the SBT-CO_2_ plot is reported in [2] and [3], and here it is taken as the exhaled volume corresponding to the point of inflection on the SBT-CO_2_ curve. Alveolar tidal volume is the part of inhaled volume that reach the alveoli

$$V_{T}^{alv}=V_{T}-V_{D}^{aw}\eqno{\hbox{(2)}}$$

where $V_{T}^{alv}$ is alveolar tidal volume and $V_{D}^{aw}$ is airway dead space. However, not all inhaled air that reaches the gas exchanging zone takes part in the mixing process. This fractional volume in alveoli that does not contribute to gas exchange is termed alveolar dead space. Combination of alveolar dead space and airway dead space defines the total dead space of the respiratory system

**Fig. 2.**
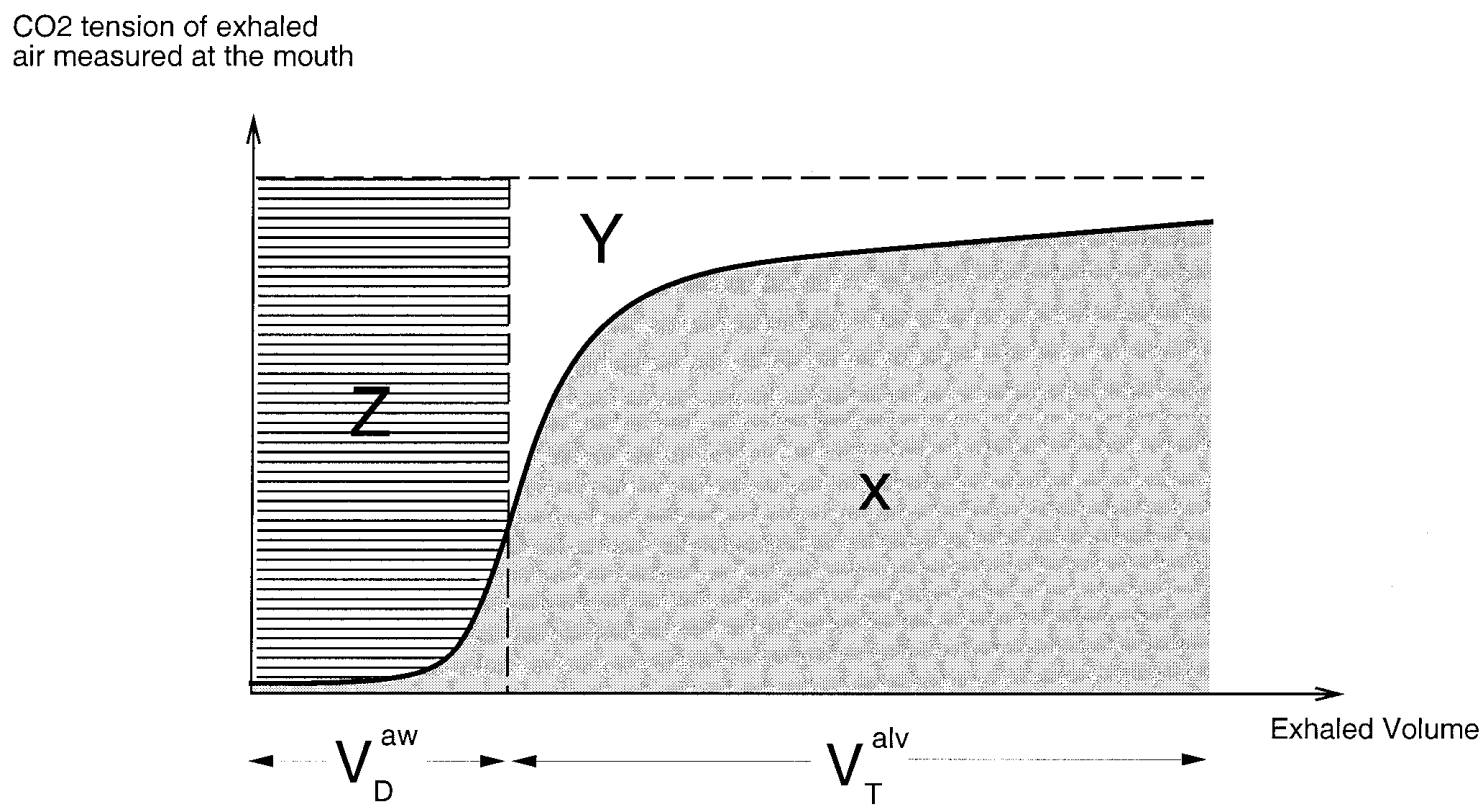
SBT-CO_2_ plot and the partition of regions $X,\,Y,$ and $Z$.

$$V_{D}=V_{D}^{alv}+ V_{D}^{aw}\eqno{\hbox{(3)}}$$

where $V_{D}^{alv}$ is alveolar dead space. The effective alveolar ventilation is the difference between alveolar tidal volume and alveolar dead space

$$V_{A}^{eff} = V_{T}^{alv}-V_{D}^{alv} = V_{T}-V_{D}\eqno{\hbox{(4)}}$$

where $V_{A}^{eff}$ is the effective alveolar ventilation.

The alveolar dead space can be related to areas of SBT-CO_2_ plot (see Fig. 2). In Fig. 2, the dashed vertical line falls through the point of inflection of the SBT-CO_2_ curve. The areas $X,\,Y$ and $Z$ represents the following.

$$\matrix{\multispan 2\hrulefill\cr \noalign{\vskip -4pt}{\hbox{Area-$X$}} & {\hbox{Efficient part of ventilation.}}\hfill\cr \noalign{\vskip 6pt}{\hbox{Area-$Y$}} & {\hbox{A defect in CO$_2$ elimination that}}\hfill\cr & {\hbox{represents wasted ventilation due to}}\hfill\cr & {\hbox{alveolar dead space.}}\hfill\cr \noalign{\vskip 6pt}{\hbox{Area-$Z$}} & {\hbox{A defect in CO$_2$ elimination that}}\hfill\cr & {\hbox{represents wasted ventilation due to}}\hfill\cr & {\hbox{airway dead space.}}\hfill\cr \noalign{\vskip -6pt}\multispan 2\hrulefill}$$

The alveolar dead space can be defined using Area-$X$ and Area-$Y$ [1], [4]

$$V_{D}^{alv}= {{{\hbox{Area-$Y$}}}\over {{\hbox{Area-$Y$}} + {\hbox{Area-$X$}}}}\, V_{T}^{alv}.\eqno{\hbox{(5)}}$$

Refer to the Appendix for a clinical trial on the measurement of airway deadspace and alveolar deadspace of a mechanically ventilated patient in the Intensive Care Unit (ICU) at the Royal Melbourne Hospital (RMH).

## III. Arterial CO_2_ Tension Estimation

This section describes two algorithms [5] that can estimate arterial CO_2_ tension of mechanically ventilated patients. The continuous estimation of arterial CO_2_ tension is based on instantaneous, correlated measurements of ventilatory flow, airway pressure, and CO_2_ tension of exhaled air measured by a capnograph. Although end tidal CO_2_ tension may approximately follow the variations in arterial CO_2_ tension, various studies have shown that utilization of end tidal CO_2_ as a noninvasive monitoring substitute for trends in arterial CO_2_ tension in critically ill patients may be misleading [6], [7]. The algorithms described here are based on the efficiency of gas mixing in the lung as oppose to an algorithm based on end tidal CO_2_ tension. The efficiency of gas exchange may be defined using Bohr's equation

$${{V_{D}}\over {V_{T}}} = 1 - {{P_{\,\overline{E}_{{\rm CO}_2}}}\over {P_{a_{{\rm CO}_2}}}} = 1 - {\hbox{efficiency}}\eqno{\hbox{(6)}}$$

where $V_{D}$ is the dead space, $V_{T}$ is the tidal volume, $P_{a_{{\rm CO}_2}}$ is the arterial CO_2_ tension, and $P_{\,\overline{E}_{{\rm CO}_2}}$ is the average CO_2_ tension of exhaled air. The term $P_{\,\overline{E}_{{\rm CO}_2}}/P_{a_{{\rm CO}_2}}$ represents how well the inhaled tidal volume is used in the lungs for the purpose of gas exchange, and hence the term may be referred to as the efficiency of gas mixing

$${\hbox{efficiency}} = {{P_{\,\overline{E}_{{\rm CO}_2}}}\over {P_{a_{{\rm CO}_2}}}}.\eqno{\hbox{(7)}}$$

Experiments carried out on human subjects [5], [8] have found that the efficiency of gas mixing stays relatively constant for various levels of ventilation. Hence, if the efficiency of gas mixing is calculated upon performing a blood test and assumed constant in an individual patient for subsequent breaths following the blood test, the only unknown in (7) is the blood CO_2_ tension, provided that the average partial pressure of CO_2_ in exhaled air is known. Using basic gas laws, $P_{\,\overline{E}_{{\rm CO}_2}}$ can be expressed as follows:

$$P_{\,\overline{E}_{{\rm CO}_2}}={{1}\over {V_T}}\, \int_{0}^{T} \left({{P_{{\rm CO}_2}(t)(P_{B}-P_{{\rm H}_2{\rm O}})}\over {P(t)+P_{B}-P_{{\rm H}_2{\rm O}}}}\right)\mathdot{V}(t)\, dt\eqno{\hbox{(8)}}$$

where $P_{{\rm CO}_2} (t)$ is the partial pressure of CO_2_ in exhaled air, $P(t)$ is mouth pressure, $\mathdot{V} (t)$ is the flow, $V_T$ is the tidal volume, $T$ is the exhalation time, $P_{B}$ is the barometric pressure, and $P_{{\rm H}_2{\rm O}}$ is the saturated water vapor pressure.

It should be noted that the accuracy of estimation can be affected by possible variations in efficiency of gas mixing that may occur. The problem of changes in efficiency can be overcome by incorporating a monitoring module to detect such changes and to request the operator to perform a new blood test to calculate the new efficiency of gas mixing thereby improving the accuracy of CO_2_ estimation. The efficiency of gas mixing is considered to change if any of the following events occur three times.

• If minute volume increases by 800 ml/min or more and end-tidal CO_2_ tension^3.^^3.^End-tidal CO_2_ tension is the partial pressure of CO_2_ in a sample of exhaled air taken at the end of exhalation. does not drop by at least 2 mmHg.
• If minute volume decreases by 800 ml/min or more and end-tidal CO_2_ tension does not increase by at least 2 mmHg.
• If minute volume increases by 800 ml/min or more and estimated CO_2_ tension does not drop by at least 2 mmHg.
• If minute volume decreases by 800 ml/min or more and estimated CO_2_ tension does not increase by at least 2 mmHg.
• If inspiratory time changes by more than 0.5 s.
• If peak airway pressure changes more than 10 cm H_2_ O.
• If previous PEEP^4.^ ^4.^PEEP stands for positive end expiratory pressure. is less than 10 cm H_2_ O and changes by more than 5 cm H_2_ O.
• If previous PEEP is greater than or equal to 10 cm H_2_ O and changes by more than 2 cm H_2_ O.

Equations (7) and (8) together with a monitoring module for detecting changes in efficiency of gas mixing provide a framework for estimating arterial CO_2_ tension. Algorithm 1 outlines a technique for estimating arterial CO_2_ tension.

$$\boxaround{\matrix{\noalign{\vskip 4pt}{\hbox{Algorithm 1}}\cr \noalign{\vskip 8pt}1.0\qquad {\hbox{Step one: Based on initial simultaneous}}\hfill\cr \hskip 80pt P_{a_{{\rm CO}_2}} {\hbox{ and SBT-CO}}_2 {\hbox{ trace}}\hfill\cr \noalign{\vskip 6pt}\hskip 34pt 1.1 {\hbox{ Do a blood test and measure $P_{a_{{\rm CO}_2}}$}}\hfill\cr \hskip 34pt 1.2 {\hbox{ Use (8) to calculate $P_{\,\overline{E}_{{\rm CO}_2}}$}}\hfill\cr \hskip 34pt 1.3 {\hbox{ Calculate efficiency using Bohr{'}s}}\hfill\cr \hskip 50pt {\hbox{equation}}\hfill\cr \noalign{\vskip 6pt}{\hbox{efficiency}} = {\displaystyle {{P_{\,\overline{E}_{{\rm CO}_2}}}\over {P_{a_{{\rm CO}_2}}}}}\cr \noalign{\vskip 6pt}2.0\qquad {\hbox{Step two: Based on subsequent SBT-CO$_2$}}\hfill\cr \hskip 80pt {\hbox{trace without $P_{a_{{\rm CO}_2}}$ measurement}}\hfill\cr \noalign{\vskip 6pt}\hskip 34pt 2.1 {\hbox{ Use (8) to calculate $P_{\,\overline{E}_{{\rm CO}_2}}$}}\hfill\cr \hskip 34pt 2.2 {\hbox{ Estimate CO$_2$ tension using Bohr{'}s}}\hfill\cr \hskip 50pt {\hbox{equation}}\hfill\cr \noalign{\vskip 6pt}{\hbox{Estimated }} P_{a_{{\rm CO}_2}} = {\displaystyle {{ P_{\,\overline{E}_{{\rm CO}_2}}}\over {{\hbox{efficiency}}}}}\cr \noalign{\vskip 6pt}3.0\qquad {\hbox{Step three: Applicable to measurement in}}\hfill\cr \hskip 85pt {\hbox{Step two}}\hfill\cr \noalign{\vskip 6pt}\hskip 34pt 3.1 {\hbox{ Has the efficiency changed?}}\hfill\cr \hskip 52pt {\hbox{Yes: Go to step one}}\hfill\cr \hskip 52pt {\hbox{No: Go to step two}}\hfill\cr \noalign{\vskip 4pt}}}$$

Efficiency of gas mixing can also be defined using the areas of SBT-CO_2_ curve [9]

$${\hbox{Efficiency}} = 1-{{V_{D}^{alv}}\over {V_{T}^{alv}}} = {{{\hbox{Area-$X$}}}\over {{\hbox{Area-$X$}} + {\hbox{Area-$Y$}}}}.\eqno{\hbox{(9)}}$$

The algorithm proposed by [5] for CO_2_ estimation is based on the efficiency expression derived by Fletcher. The method of estimation is shown in Algorithm 2.

$$\boxaround{\matrix{\noalign{\vskip 4pt}{\hbox{Algorithm 2}}\cr \noalign{\vskip 8pt}1.0\qquad {\hbox{Step one: Based on initial simultaneous}}\hfill\cr \hskip 80pt P_{a_{{\rm CO}_2}} {\hbox{ and SBT-CO}}_2 {\hbox{ trace}}\hfill\cr \noalign{\vskip 6pt}\hskip 34pt 1.1 {\hbox{ Do a blood test and measure $P_{a_{{\rm CO}_2}}$}}\hfill\cr \hskip 34pt 1.2 {\hbox{ Calculate Area-$X$ and Area-$Y$ using}}\hfill\cr \hskip 50pt {\hbox{SBT-CO$_2$ plot}}\hfill\cr \hskip 34pt 1.3 {\hbox{ Calculate efficiency using}}\hfill\cr \noalign{\vskip 6pt}{\hbox{efficiency}} = {\displaystyle {{ {\hbox{Area-$X$}} }\over { {\hbox{Area-$X$}} + {\hbox{Area-$Y$}}}}}\cr \noalign{\vskip 6pt}2.0\qquad {\hbox{Step two: Based on subsequent SBT-CO$_2$}}\hfill\cr \hskip 80pt {\hbox{trace without $P_{a_{{\rm CO}_2}}$ measurement}}\hfill\cr \noalign{\vskip 6pt}\hskip 34pt 2.1 {\hbox{ Calculate Area-$X$ from SBT-CO$_2$ plot}}\hfill\cr \hskip 34pt 2.2 {\hbox{ Calculate $V_T^{alv}$ from SBT-CO$_2$ plot}}\hfill\cr \hskip 34pt 2.3 {\hbox{ Estimate Area-$Y$ using}}\hfill\cr \noalign{\vskip 6pt}{\hbox{Estimated Area-$Y$}} = \left({\displaystyle {{1 - {\hbox{Efficiency}}}\over {{\hbox{Efficiency}}}}}\right) {\hbox{Area-$X$}}\cr \noalign{\vskip 6pt}\hskip 34pt 2.4 {\hbox{ Estimate Arterial CO$_2$ tension using the}}\hfill\cr \hskip 48pt {\hbox{ following equation}}\hfill\cr \noalign{\vskip 6pt}{\hbox{Estimated }} P_{a_{{\rm CO}_2}} = {\displaystyle {{{\hbox{Area-$X$ $+$ Estimated Area-$Y$}}}\over {V_T^{alv}}}}\cr \noalign{\vskip 6pt}3.0\qquad {\hbox{Step three: Applicable to measurement in}}\hfill\cr \hskip 85pt {\hbox{Step two}}\hfill\cr \noalign{\vskip 6pt}\hskip 34pt 3.1 {\hbox{ Has the efficiency changed?}}\hfill\cr \hskip 52pt {\hbox{Yes: Go to step one}}\hfill\cr \hskip 52pt {\hbox{No: Go to step two}}\hfill\cr \noalign{\vskip 4pt}}}$$

It should be noted that the efficiency expression resulting from Fletcher's equation includes only alveolar dead space and alveolar tidal volume as opposed to total dead space and total tidal volume incorporated in the efficiency expression resulting from Bohr's equation. It has been reported that efficiency defined by Fletcher's equation is less sensitive to changes in ventilation than the efficiency defined using Bohr's equation [5]. Hence, it may be expected that incorporating the efficiency expression derived using Fletcher's equation for arterial CO_2_ estimation produces more accurate readings of arterial CO_2_ than incorporating the efficiency expression derived using Bohr's equation. The algorithm implemented by [5] claims an accuracy of ±4 mmHg.

**Fig. 3.**
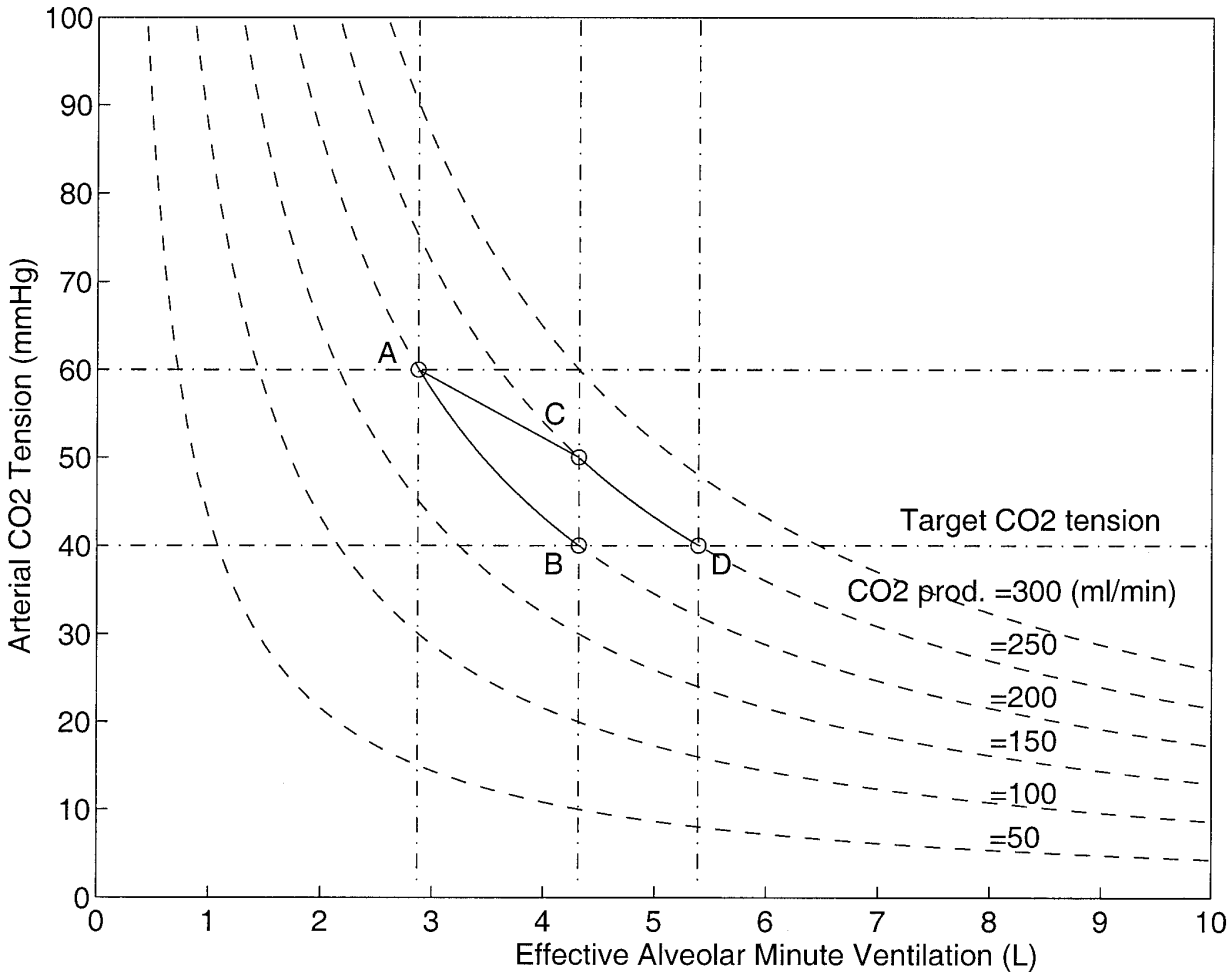
One-step-ahead control performance.

## IV. Control of $P_{a_{{\rm CO}_2}}$ By Regulating $V_{A}^{\rm eff}$

The level of arterial CO_2_ tension is dependent on the CO_2_ production in cells and the magnitude of effective alveolar ventilation. A disturbance in CO_2_ production or the magnitude of effective alveolar ventilation will alter the level of arterial CO_2_ tension. The relationship between these variables are given by the alveolar air equation [10]

$$P_{a_{{\rm CO}_2}} = {{0.863 * \mathdot{V}_{{\rm CO}_2}}\over {\mathdot{V}_{A}^{eff}}}.\eqno{\hbox{(10)}}$$

Fig. 3 shows the inverse relationship between the blood carbon dioxide tension and the effective alveolar minute ventilation for various CO_2_ production values.

**Fig. 4.**
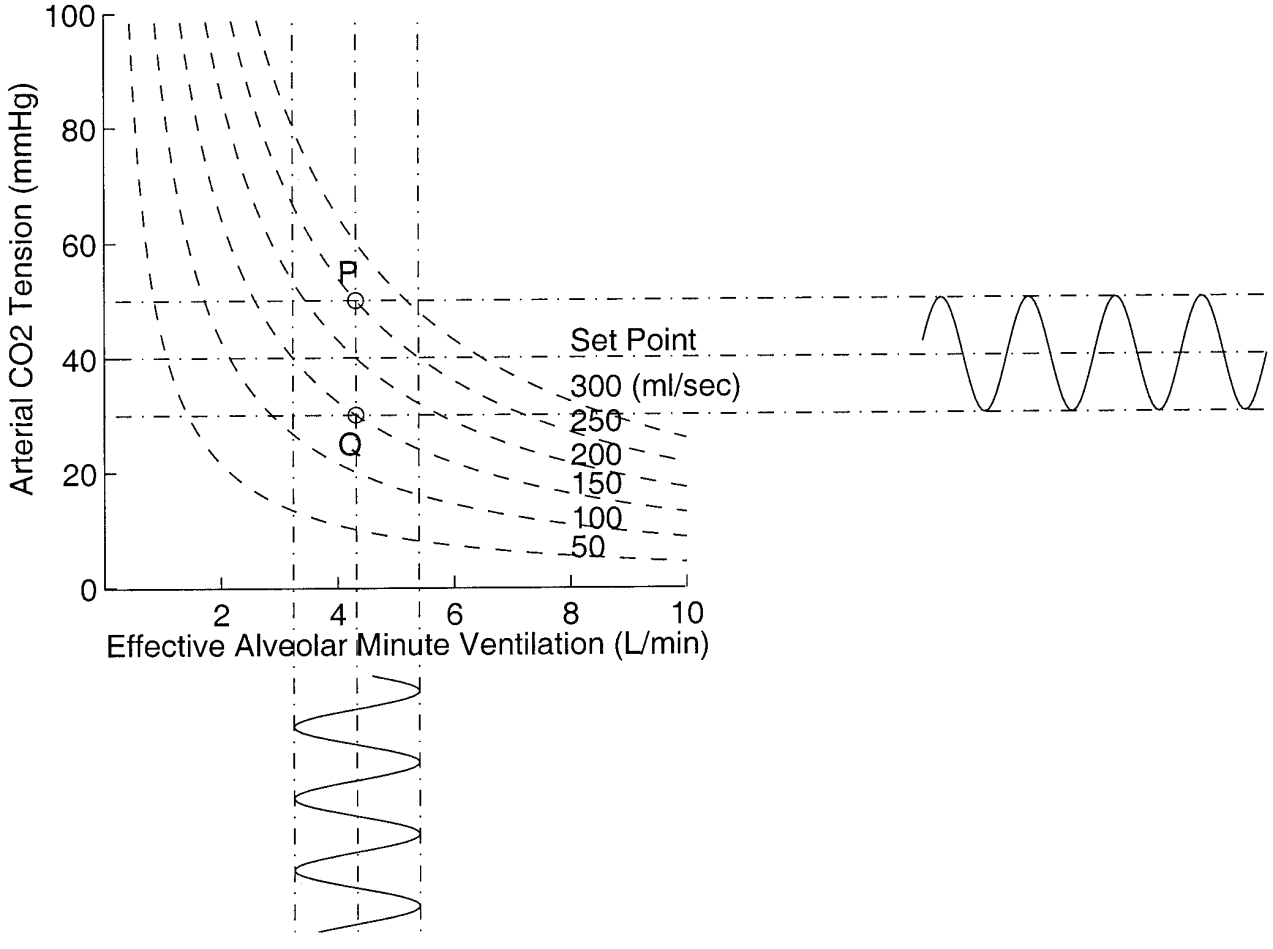
Oscillations of CO_2_ production, arterial CO_2_, and effective alveolar minute ventilation.

A one-step-ahead control strategy may be adopted in the regulation of blood CO_2_ tension. A one-step-ahead controller by definition attempts to push the control variable (i.e., $P_{a_{{\rm CO}_2}}$) from the current value to the desired value in one step. The CO_2_ controller takes corrective action every five minutes, which is also the sampling time of the controller. The performance of the controller can be explained by referring to the details of Fig. 3.

A patient with an unacceptable blood CO_2_ tension of 60 mmHg with an alveolar minute ventilation of 2.8 l/min and CO_2_ production of 200 ml/min can be represented at point A on the diagram. The controller in an attempt to push the patient's blood CO_2_ tension from the present value to the desired value of 40 mmHg calculates a desired alveolar ventilation of 4.3 l. If the CO_2_ production remains constant until the next sample (i.e., 5 min into the future), the patient descends down line AB and reaches the desired CO_2_ level in one step. The one-step-ahead control law can be written as

$${}^{new}V_{A}^{\rm eff} = {}^{\rm current}V_{A}^{\rm eff}\, {{{}^{\rm current}P_{a_{{\rm CO}_2}}}\over {{}^{\rm target}P_{a_{{\rm CO}_2}}}}\eqno{\hbox{(11)}}$$

where ${}^{\rm current}V_{A}^{\rm eff}$ is the current effective alveolar ventilation, ${}^{\rm current}P_{a_{{\rm CO}_2}}$ is the current arterial CO_2_ tension, ${}^{\rm target}P_{a_{{\rm CO}_2}}$ is the desired arterial CO_2_ tension, and ${}^{\rm new}V_{A}^{\rm eff}$ is the calculated new effective alveolar ventilation to correct the current arterial CO_2_ tension. The above control strategy is derived using (10) and assuming that $\mathdot{V}_{{\rm CO}_2}$ is constant between sampling periods. However, if the CO_2_ production changes (e.g., it increases from 200 mL/min to 250 mL/min), the patient instead of descending from point A to point B moves from point A to point C. In this situation, the target value is not reached in one step due to the disturbance in the CO_2_ production; the controller again attempts to push the patient from point C to the new desired point D by calculating a new alveolar ventilation of 5.4 L. It is clear that desired performance of the controller can be guaranteed even under sudden changes in CO_2_ production, provided it remains stable after the change. However, if the CO_2_ production is unstable and oscillates about a mean CO_2_ production value of $\mathdot{V}_{{\rm CO}_2}$ with an amplitude of $\Delta\mathdot{V}_{{\rm CO}_2}$, then from (10) it is clear that blood CO_2_ tension oscillates about the desired CO_2_ tension with an amplitude of $\Delta P_{a_{{\rm CO}_2}}$ where

$$\Delta P_{a_{{\rm CO}_2}} = 0.863\, {{\Delta\mathdot{V}_{{\rm CO}_2}}\over {\mathdot{V}_{A}^{eff}}}\eqno{\hbox{(12)}}$$

provided that alveolar ventilation is unchanged. Fig. 4 shows this scenario, as the CO_2_ production oscillates between 150 to 250 mL/min for a constant effective alveolar minute ventilation of 4.3 L represents an oscillatory movement of a point between $P$ and $Q$. It is clear that arterial CO_2_ tension will oscillate between 30 to 50 mmHg as shown in the horizontal oscillations. If the magnitude and direction of CO_2_ production oscillations are

**Fig. 5.**
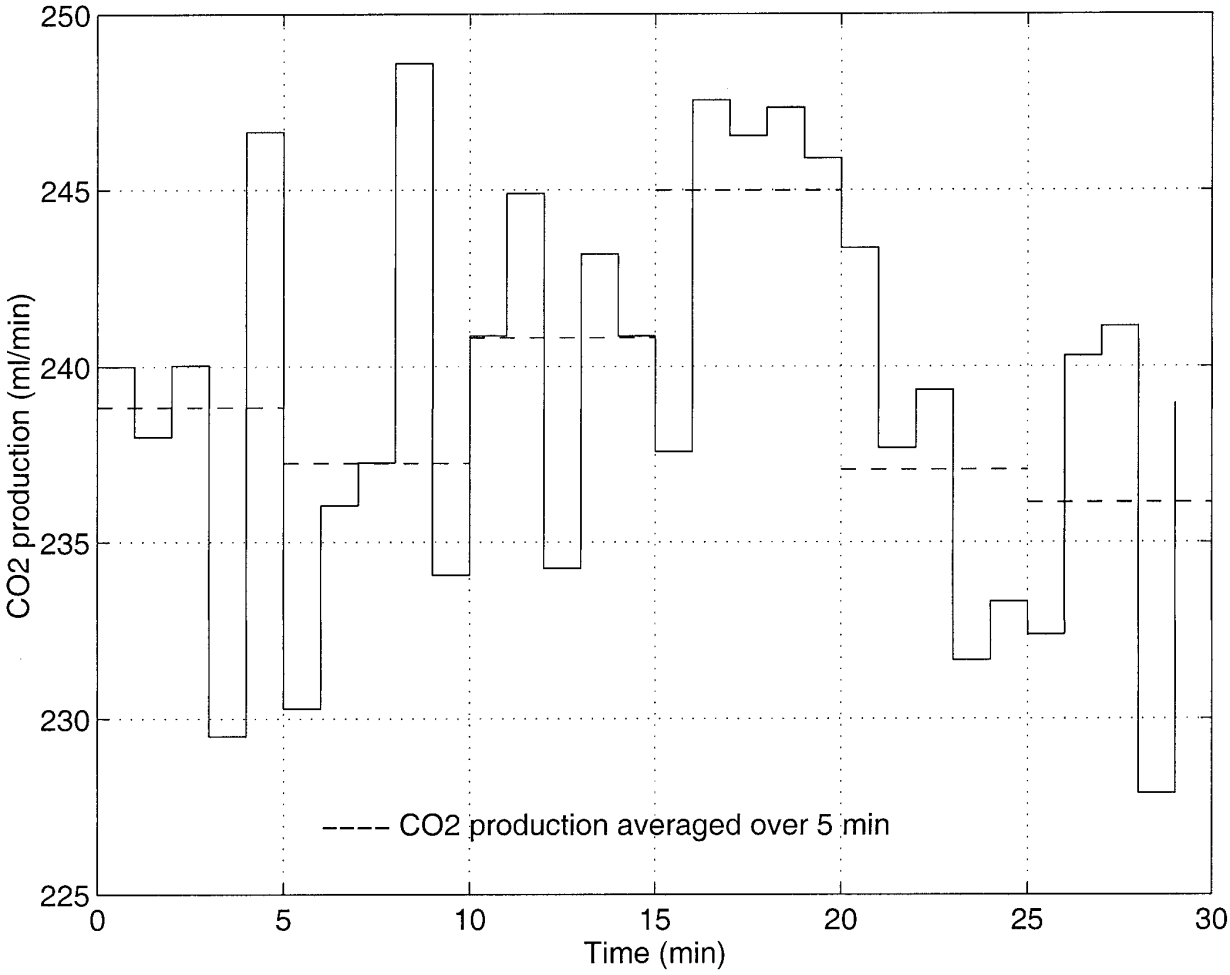
Variation of carbon dioxide production over time. The online estimation was performed in ICU at RMH.

known, the controller can then suppress the oscillations in CO_2_ tension by oscillating the alveolar minute ventilation with an amplitude of $\Delta \mathdot{V}_{A}^{eff}$ where

$$\Delta \mathdot{V}_{A}^{eff}=\mathdot{V}\, {{\Delta \mathdot{V}_{{\rm CO}_2}}\over {\mathdot{V}_{A}^{eff}}}.\eqno{\hbox{(13)}}$$

The suppression only occurs when the CO_2_ production oscillations are in phase with the effective alveolar minute ventilation oscillations. The vertical oscillations in Fig. 4 indicates how effective alveolar minute ventilation should oscillate in order to suppress the CO_2_ tension oscillations. If the CO_2_ production oscillations and the $V_{A}^{\rm eff}$ oscillations are out of phase then from (10) it is clear that the blood CO_2_ tension will oscillate with amplitude

$$\Delta P_{a_{{\rm CO}_2}} = 0.863\, {{\Delta\mathdot{V}_{{\rm CO}_2}}\over {\mathdot{V}_{A}^{\rm eff}}}\, {{2\mathdot{V}_{{\rm CO}_2}}\over {\mathdot{V}_{{\rm CO}_2}+\Delta\mathdot{V}_{{\rm CO}_2}}}\eqno{\hbox{(14)}}$$

which is greater than the CO_2_ tension oscillations when there is no CO_2_ control action present.

Since the disturbance in CO_2_ production depends on body metabolism, it cannot be predicted, though it can be continuously measured. Controlling CO_2_ tension when oscillatory disturbances are present in CO_2_ production may intensify the existing oscillations. When consistent oscillations are present in the CO_2_ production, the best performance the controller can achieve is by regulating the CO_2_ tension using a running mean CO_2_ production value instead of regulating it using the single CO_2_ production value at the time of sampling.

From the above discussion it is clear that to control the arterial CO_2_ level, it is necessary that the assumption the CO_2_ production is relatively constant be valid and regulation of effective alveolar minute ventilation be possible. Fig. 5 shows a typical variation of CO_2_ production over a period of 30 min. Here the average CO_2_ production varies within a band of 10 mL and from (12)it is clear that this will cause the arterial CO_2_ tension to oscillate with an amplitude $4.3/V_{A}^{\rm eff}$ provided that effective alveolar minute ventilation is left unaltered. For $V_{A}^{\rm eff}$ in the range of 4 to 8 L, the expected arterial CO_2_ tension oscillations have amplitudes ranging from 1.1 mmHg to 0.5 mmHg. Since the target $P_{a_{{\rm CO}_2}}$ tension is generally set around 40 mmHg, a control system that can regulate the $P_{a_{{\rm CO}_2}}$ level with in a band of 1.1 mmHg can be considered entirely acceptable. Such typical variation in CO_2_ production is, therefore, not a performance limiting factor of the control system.

**Fig. 6.**
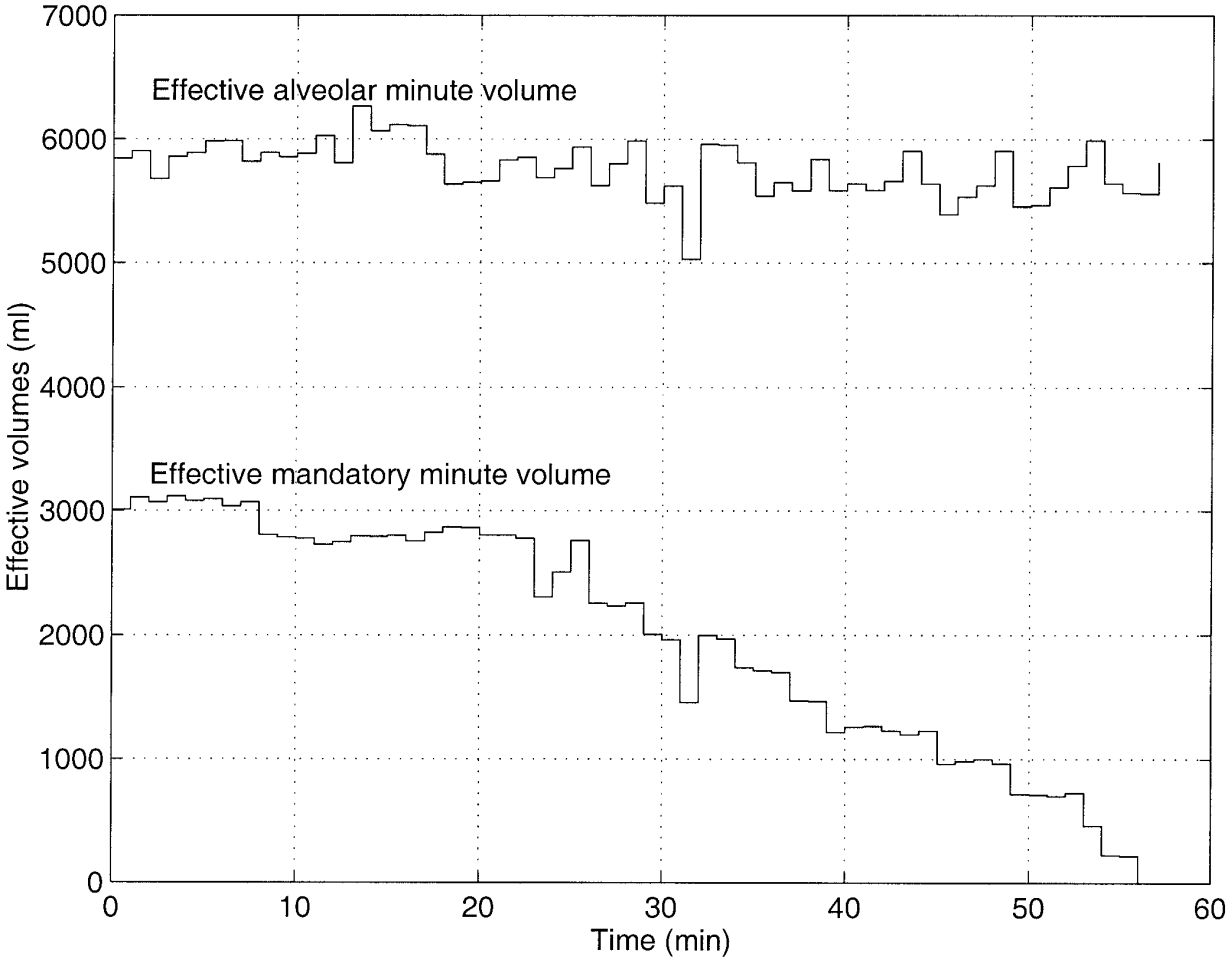
Pattern of changes in effective mandatory minute volume and the corresponding changes in effective alveolar minute ventilation. All measurements were carried out at RMH.

The minute ventilation for a mechanically ventilated patient depends on the patient's own respiratory drive and the number of mandatory breaths the operator specifies. The mandatory breaths contribute to a certain mandatory minute ventilation and the patient's respiratory drive contributes to a certain spontaneous minute volume. Both the spontaneous and mandatory minute volumes contain respiratory dead space volume, and subtraction of the two gives the effective minute volume carried by spontaneous and mandatory breaths. The controlling of $P_{a_{{\rm CO}_2}}$ assumes that regulating $V_{A}^{\rm eff}$ is possible. The effective alveolar minute ventilation is composed of the effective minute volume contributed by the spontaneous breaths (i.e., $V_{sp}^{\rm eff}$) and also the effective minute volume contributed by the mandatory breaths (i.e., $V_{\rm man}^{\rm eff}$)

$$V_{A}^{\rm eff}=V_{\rm man}^{\rm eff}+V_{sp}^{\rm eff}.\eqno{\hbox{(15)}}$$

The regulation of $V_{A}^{\rm eff}$ can only be done by adjusting $V_{\rm man}^{\rm eff}$. To be able to set any desired $V_{A}^{\rm eff}$ by adjusting $V_{\rm man}^{\rm eff}$ it is necessary that these two quantities are directly related, any changes to $V_{\rm man}^{\rm eff}$ must be reflected in $V_{A}^{eff}$. Fig. 6 shows an example of changes in $V_{\rm man}^{\rm eff}$ and the corresponding changes in $V_{A}^{\rm eff}$. From this it is clear that the variations in $V_{A}^{\rm eff}$ does not necessarily follow the those in $V_{\rm man}^{\rm eff}$. A change in $V_{\rm man}^{\rm eff}$ may alter both the $V_{sp}^{\rm eff}$ and $V_{A}^{\rm eff}$. There is no direct relationship between $V_{\rm man}^{\rm eff}$ and $V_{A}^{\rm eff}$, since alterations in $V_{\rm man}^{\rm eff}$ does not get reflected in $V_{A}^{\rm eff}$. Hence, controlling the $P_{a_{{\rm CO}_2}}$ level about a target value is not possible in such circumstances.

However, if a patient receives a certain number of mandatory breaths, then this ensures that the patient is guaranteed a certain level of effective alveolar minute ventilation, i.e.,

$$V_{A}^{\rm eff}\geq V_{\rm man}^{\rm eff}.\eqno{\hbox{(16)}}$$

From (10) and (16), the following can be written:

$$P_{a_{{\rm CO}_2}}={{0.832 \mathdot{V}_{{\rm CO}_2}}\over {V_{A}^{\rm eff}}} \leq {{0.832 \mathdot{V}_{{\rm CO}_2}}\over {V_{\rm man}^{\rm eff}}} = P_{a_{{\rm CO}_2}}^{\max}.\eqno{\hbox{(17)}}$$

From (17), it is clear that using $V_{\rm man}^{\rm eff}$ as the control signal and (11) as the control law can only maintain arterial CO_2_ level below a certain maximum level (i.e., $P_{a_{{\rm CO}_2}}^{\max}$). Similarly, (16) and (17) can be rewritten for a patient ventilated in the MMV mode as

$$V_{A}^{\rm eff}\geq {\hbox{MMV}} - \mathdot{V}_{D}\eqno{\hbox{(18)}}$$

where $\mathdot{V}_{D}$ is the deadspace volume corresponding to MMV and

$$P_{a_{{\rm CO}_2}}= {{0.832 \mathdot{V}_{{\rm CO}_2}}\over {V_{A}^{\rm eff}}} \leq {{0.832 \mathdot{V}_{{\rm CO}_2}}\over {{\hbox{MMV}} - \mathdot{V}_{D}}} = P_{a_{{\rm CO}_2}}^{\max}.\eqno{\hbox{(19)}}$$

It is clear that in this situation using the MMV setting as the control signal and (11) as the control law, again can only maintain arterial CO_2_ tension below a certain maximum level (i.e., $P_{a_{{\rm CO}_2}}^{\max}$). Hence, it may be stated that, while arterial CO_2_ tension cannot be controlled about a set target level, it is possible to maintain it below a certain maximum level.

**Fig. 7.**
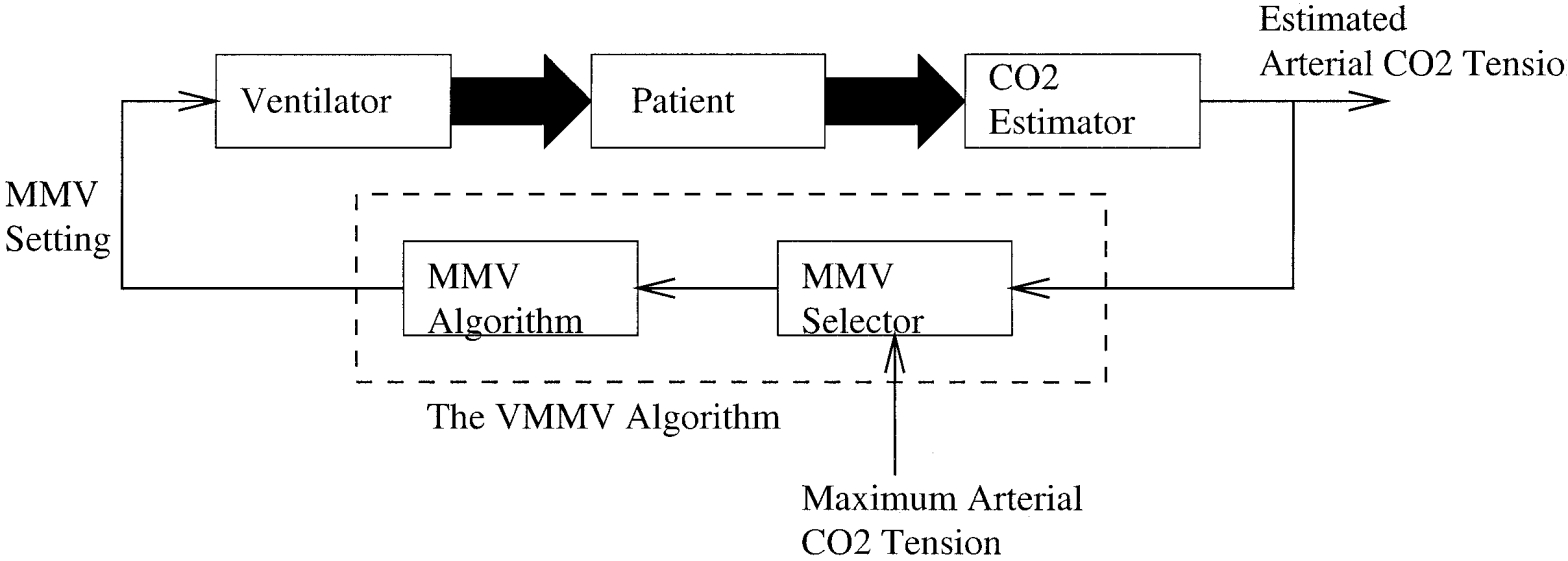
Implementation of the VMMV algorithm.

## V. VMMV Algorithm

To maintain arterial CO_2_ tension below $P_{a_{{\rm CO}_2}}^{\max}$, $V_{A}^{\rm eff}$ must be regulated according to (11) periodically. The periodic implementation of $V_{A}^{\rm eff}$ can be done in the MMV mode by periodically selecting a MMV setting as follows:

$${}^{\rm new}{\hbox{MMV}}= {}^{\rm new} V_{A}^{\rm eff} + {}^{\rm new}\mathdot{V}_{D}\eqno{\hbox{(20)}}$$

where ${}^{\rm new}\mathdot{V}_{D}$ is the deadspace volume corresponding to ${}^{\rm new}V_{A}^{\rm eff}$. The new minute dead space volume can be estimated by assuming that minute dead space volume varies proportionally to minute volume [8]

$${{{}^{\rm new}\mathdot{V}_{D}}\over {{}^{\rm new}\mathdot{V}_{D} + {}^{\rm new}V_{A}^{\rm eff}}} = {{{}^{\rm current}\mathdot{V}_{D}}\over {{}^{\rm current}\mathdot{V}_{D} + {}^{\rm current}V_{A}^{\rm eff}}}.\eqno{\hbox{(21)}}$$

Therefore

$$\eqalignno{{}^{\rm new} \mathdot{V}_{D} =\,& {}^{\rm current}\mathdot{V}_{D} \left({{{}^{\rm new}V_{A}^{\rm eff}}\over {{}^{\rm current} V_{A}^{\rm eff}}}\right)\cr \noalign{\vskip 4pt}=\,& {}^{\rm current}\mathdot{V}_{D} \left({{P_{a_{{\rm CO}_2}}^{\rm current}}\over {P_{a_{{\rm CO}_2}}^{\max}}}\right).&{\hbox{(22)}}}$$

Using (20) and (22) the expression for ${}^{\rm new}{\hbox{MMV}}$ can be rewritten as

$$\eqalignno{{}^{\rm new}{\hbox{MMV}} =\,& {}^{\rm new} V_{A}^{\rm eff} + {}^{\rm new}\mathdot{V}_{D}\cr \noalign{\vskip 4pt}=\,& \left({{P_{a_{{\rm CO}_2}}^{\rm current}}\over {P_{a_{{\rm CO}_2}}^{\max}}}\right) \left({}^{\rm current}V_{A}^{eff} + {}^{\rm current} \mathdot{V}_{D}\right)\cr \noalign{\vskip 4pt}=\,& {\hbox{MV}} \left({{P_{a_{{\rm CO}_2}}^{\rm current}}\over {P_{a_{{\rm CO}_2}}^{\max}}}\right).&{\hbox{(23)}}}$$

Fig. 7 is a schematic of a patient ventilated in VMMV mode. Arterial CO_2_ tension (rather than minute ventilation) serves as the input to the VMMV algorithm. The periodic MMV selection is based on (23). Based on the arterial CO_2_ tension and minute ventilation, both averaged over 5–min intervals, the MMV selector outputs a new MMV setting every 5 min. The following is a clinical trial of a patient ventilated in the new VMMV mode.

A patient who had been receiving mechanical ventilation for the past 72 hs was chosen to trial on the VMMV mode. The patient was on a rate of 12 volume control breaths per minute, each carrying a tidal volume of 600 mL. A blood test was performed 4 min into the trial, and it revealed an arterial CO_2_ tension of 41 mmHg. Based on the arterial CO_2_ tension in this minute, a blood gas efficiency of 0.646 was calculated (refer to Section III for details of this calculation). The VMMV mode was not engaged until the 21st minute, until then only the CO_2_ estimator was in operation. Within this time span, arterial CO_2_ tension varied between 38.9–41.1 mmHg. When the VMMV mode was engaged a maximum arterial CO_2_ tension setting of 41 mmHg was chosen as the input to the algorithm.

**Fig. 8.**
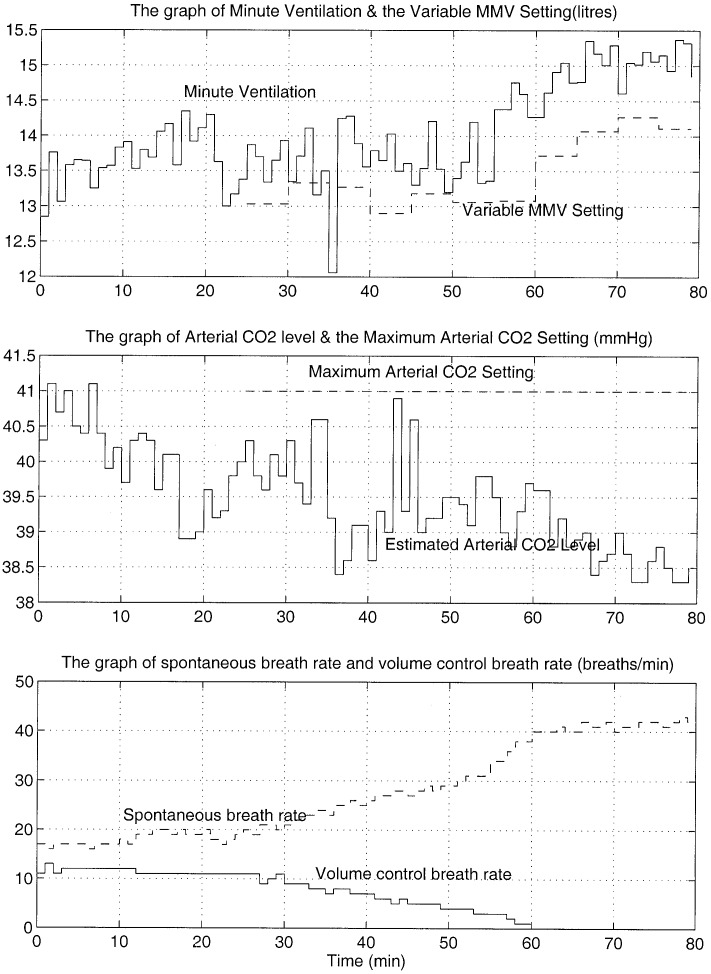
Variations of minute volume, the MMV setting, the arterial CO_2_ tension, spontaneous, and volume control breaths per minute. This VMMV trial was carried out in ICU at RMH.

The minute volume, MMV settings, estimated arterial CO_2_ tension, and spontaneous and volume control breaths per minute are shown in Fig. 8. By the 61st minute, the patient was receiving no volume control breaths and was breathing spontaneously. The minute ventilation then remained above the MMV setting constantly for another 20 min at which point the trial was terminated. A blood gas performed in the 78th minute revealed an arterial CO_2_ tension of 36.7 mmHg. The estimated arterial CO_2_ tension was 1.6 mmHg different from the value obtained from gas analysis. It is noted that throughout the trial, the MMV setting has changed at every 5-min interval, and the arterial CO_2_ tension has remained below the set maximum limit. The new ventilation mode has demonstrated the ability to encourage patient's spontaneous breathing while ensuring that the arterial CO_2_ tension remained below the prescribed maximum settings as determined by the clinician.

The control system cannot raise the arterial CO_2_ tension above the level achieved during spontaneous ventilation. In situations where arterial CO_2_ tension needs to be increased, patient's spontaneous ventilation needs to be suppressed through sedative medication allowing the mechanical ventilator to “take over” in regulating effective alveolar ventilation and thereby controlling the arterial CO_2_ tension. Tighter control of arterial CO_2_ tension is possible if spontaneous breathing is suppressed and ventilation is controlled entirely by a ventilator.

## VI. Conclusion

**Fig. 9.**
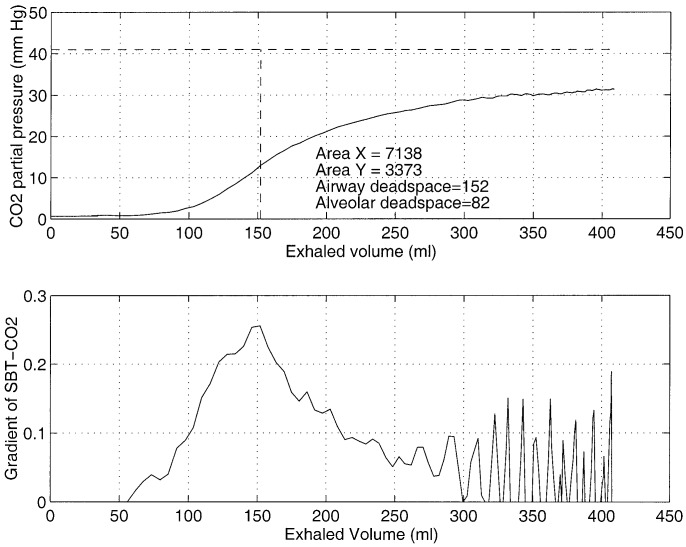
SBT-CO_2_ plot and the gradient of SBT-CO_2_. The CO_2_ partial pressure signal was obtained from a Hewlett Packard 78 356A capnograph and the flow signal was obtained from a PB-7200a ventilator. The flow signal was integrated to obtain volume. The measurements were made in ICU at RMH.

When a patient is ventilated in the VMMV mode, the clinician decides the maximum arterial CO_2_ level. Once this decision is made, the ventilation scheme calculates a new MMV setting every 5 min (hence the name variable MMV) to ensure that arterial CO_2_ level stays below the set limit. The ventilation mode while providing all features of the MMV mode can ensure that arterial CO_2_ remains below a set limit.

## Appendix

To form a SBT-CO_2_ trace, it is necessary to access the inspiratory flow waveform and CO_2_ partial pressure of exhaled air. The inspiratory flow waveform was accessed from an analog port in the PB-7200a ventilator and the CO_2_ partial pressure of exhaled air was accessed from an analog port of the HP-78 356A capnograph. Both analog signals were sampled at a frequency of 100 Hz and processed in an IBM-386 PC. The top graph of Fig. 9 shows a SBT-CO_2_ trace. The gradient of this curve is shown in the lower graph of Fig. 9. The maximum peak in the gradient of SBT-CO_2_ plot corresponds to the point of inflection of the SBT-CO_2_ plot. The maximum peak in the gradient of SBT-CO_2_ plot in this patient occurs when the exhaled volume is 152 ml. This point corresponds to the point of inflection of the SBT-CO_2_ plot and, therefore, represents the airway dead space. The tidal volume of this breath is 408 ml and hence the alveolar tidal volume is 408 − 152 = 256 ml. A blood test performed within this time revealed an arterial CO_2_ tension of 41 mmHg. The Area-$X$ and Area-$Y$ for this breath was calculated to be 7138 ml mmHg and 3373 ml mmHg, respectively. Using (5) alveolar dead space is (256 × 3373)/(7138 + 3377) $= 82$ mL. The total dead space of the respiratory system is the sum of alveolar dead space and airway dead space, 82 + 152 = 234 mL.

## References

[1] Fletcher R., Jonson B., Cumming G. and Brew J., “The concept of deadspace with special reference to the single breath test,” Brit. J. Anaesth., pp. 74–90, vol. 53, 1981. 10.1093/bja/53.1.77 6779846

[2] Flower W. S., “Lung function studies,” Amer. J. Physiol., p. 405, vol. 154, 1948. 18101134 10.1152/ajplegacy.1948.154.3.405

[3] Nunn J. F., Appl. Respiratory Physiol., London, U.K.: Butterworths, p. 178 1977.

[4] Fletcher R., The single breath test for carbon dioxide, Ph.D. dissertation Univ. Lund Lund, Sweden, 1986.

[5] Law E. B., An intelligent P_a CO 2_ estimator and controller for ventilation control, Masters thesisElect. Eng. Dept., Univ. Melbourne Melbourne, Australia, 1990.

[6] Hoffman R. A., Krieger B. P., Kramer M. R., Segel S., Bizousky F., Gazeroglu H. and Sackner M. A., “End-tidal carbon dioxide in critically ill patients during changes in mechanical ventilation,” Amer. Rev. Respir. Dis., pp. 1265–1268, vol. 140, 1989. 2510564 10.1164/ajrccm/140.5.1265

[7] Raemer D. B., Francis D., Philip J. H. and Grabel R. A., “Variation in PCO_2_ between arterial blood and peak expired gas during anesthesia,” Anesth. Analg., pp. 1065–1069, vol. 62, 1983. 6418028

[8] Nunn J. F., “Elimination of carbon dioxide by the lung,” Anesthesiology, pp. 620–633, vol. 21, no. 6, 1960. 13729910 10.1097/00000542-196011000-00006

[9] Fletcher R., “Deadspace, invasive and noninvasive,” Brit. J. Anaesth., pp. 102–107, vol. 57, 1985. 10.1093/bja/57.3.245 3919744

[10] Cade J. F. and Pain M. C., Essentials of Respiratory Medicine, Oxford, U.K.: Blackwell, 1988.

